# Highly Sensitive Ammonia Gas Sensor Based on MWCNTs Saturated Fe_2_O_3_ Nanograins

**DOI:** 10.1021/acs.langmuir.5c02458

**Published:** 2025-09-28

**Authors:** Mikayel Aleksanyan, Artak Sayunts, Gevorg Shahkhatuni, Zarine Simonyan, Davit Kananov, Andranik Grigoryan, Rima Papovyan, Dušan Kopecký

**Affiliations:** † Center of Semiconductor Devices and Nanotechnologies, 105430 Yerevan State University, 1 Alex Manoogian, 0025 Yerevan, Armenia; ‡ Department of Mathematics, Informatics and Cybernetics, Faculty of Chemical Engineering, 52735 University of Chemistry and Technology Prague, Technická 5, 166 28 Prague 6, Czech Republic

## Abstract

Ammonia has a wide range of applications in various branches of industry and technology; the detection of its low concentrations is a preventive method for ambient air quality pollution and a useful method for noninvasive diagnosis of kidney disease by monitoring in exhaled human breath. In this work, a high-performance ammonia sensor based on the Fe_2_O_3_/MWCNTs (multiwalled carbon nanotubes) material was produced by applying the e-beam deposition method. The morphology, composition, and chemical state of the Fe_2_O_3_/MWCNTs material were characterized by scanning electron microscopy (SEM), transmission electron microscopy (TEM), energy-dispersive X-ray spectroscopy (EDX), Raman spectroscopy (Raman), Fourier transform infrared (FTIR) spectroscopy, and X-ray photoelectron spectroscopy (XPS). The gas-sensing behavior of the sensor to various concentrations of NH_3_ was measured at 200 °C, representing excellent gas response and sophisticated performance parameters. The low detection limit of the Fe_2_O_3_/MWCNTs sensor was 3 ppm of NH_3_, corresponding to the response value of 2.7. The promising values of the response and recovery times (40 and 25 s) confirmed the high speed of ammonia detection. Thus, the suggested Fe_2_O_3_/MWCNTs material that is sensitive to low concentrations of NH_3_ can be successfully used in a new generation of ammonia alarm systems.

## Graphical abstract

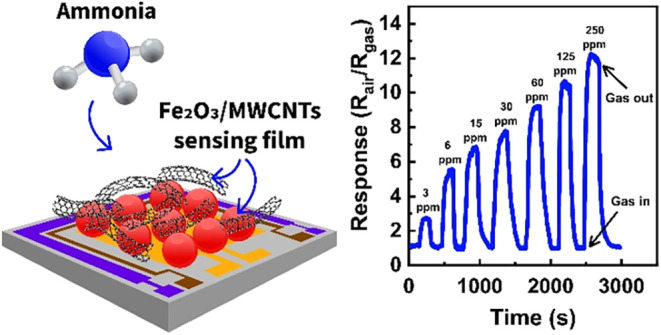

## Introduction

Rapid and accurate monitoring of ambient air quality for the prevention of health, environmental, safety, and global pollution issues is still considered a primary challenge in this fast-developing world. The most focal point here is the anomalous increase in the level of atmospheric pollution, where various types of anthropogenic sources play the main role, introducing various toxic gases into the environment.^1–7^ Among such gases, ammonia, which has a pungent smell and colorless appearance, is capable of causing unexpected health risks to human beings, such as lung damage, eye irritation, disruption of the human respiratory system, dizziness, etc.^8^ The permissible limit for ammonia is considered to be less than 50 ppm, but its widespread use in agriculture, industry, pharmaceuticals, and other fields makes it difficult to avoid exceeding this limit. Besides, ammonia emitted from animal waste on livestock farms also negatively affects the quality of dairy and meat products, the prevention of which requires constant monitoring of ammonia concentrations in the environment.^9,10^ Selective detection of low concentrations of ammonia can also serve as the basis for a noninvasive diagnostic system for kidney diseases, since its increased concentration in the exhaled air of a human being indicates the presence of kidney diseases and protein metabolic processes in the patient’s body.^11^ Thus, such a wide range of ammonia applications and the seriousness of their hazard level lead to an unjustified insistence on the need for continued development and improvement of highly effective ammonia gas sensors.

Two methods for detecting ammonia and measuring its low concentrations can be conventionally divided.^12^ The first one uses stationary laboratory installations (gas chromatography–mass spectrometry (GC-MS), selective ion flow tube–mass spectrometry (SIFT-MS), etc.) that are large and require intensive technical maintenance and laboratory care. These installations are quite inconvenient and undemanding for use in real environments for dynamically monitoring ammonia traces.^13–15^ In contrast to these, simple element-type sensors are also used, which are small in size, portable, energy-saving, and durable, such as optical, microcantilever, electrochemical, surface acoustic wave, field effect transistor, conducting polymer, and chemiresistive sensors.^16,17^ As sensors with high sensitivity and temporal stability, chemoresistive-type detectors mainly integrate metal oxide nanostructures (SnO_2_, Fe_2_O_3_, ZnO, In_2_O_3_, etc.)^18,19^ and 1*D*/2D composite materials (MWCNTs, graphene, etc.), which are increasingly improved with the development of various synthesizing methods.^20,21^ Unlike CVD (chemical vapor deposition) techniques,^22^ PVD (physical vapor deposition) methods are quite controllable, inexpensive, and the presence of a vacuum environment allows for the production of films with high purity and crystalline perfection.^23^ The e-beam deposition and magnetron sputtering methods, which occupy a unique place in the PVD variety, are quite applicable for obtaining nanostructured films from high-resistance oxide targets and carbon-based materials.^24–26^ Experimental steps for the successful deposition of iron oxide applicable to gas sensors using this method have been confirmed many times.^27,28^

There are various configurations of iron oxide in nature, of which the following three are the most well-known: magnetite (Fe_3_O_4_), maghemite (γ-Fe_2_O_3_), and hematite (α-Fe_2_O_3_).^29^ Due to its high stability and susceptibility to redox reactions, hematite is most useful in gas sensors with a pronounced blood-red color. Its band gap energy ranges from 1.9 to 2.2 eV and can be easily tuned by introducing various dopants. Its sufficient density (5.26 g/cm^3^) and high melting point (1350 °C) are favorable parameters for its high temporal stability (magnetic susceptibility: + 3586.0 × 10^–6^ cm^3^/mol, molar mass: 159.687 g·mol^–1^, refractive index: *n*_1_ = 2.91, *n*_2_ = 3.19, Std molar entropy: 87.4 J/mol·K, heat capacity: 103.9 J/mol·K, Gibbs free energy: −742.2 kJ/mol).^30–34^ Despite all this, to further improve the gas-sensing parameters, it is necessary to saturate iron oxide with various one- and two-dimensional structures, turning it into a functionalized composite composition.^35,36^ For this purpose, MWCNTs are most applicable, the introduction of which in the main material irreversibly improves the sensor’s gas sensitivity, speed, temporal stability, and reproducibility.^37^ The naturally occurring electrophysical and mechanical properties of the MWCNTs are attributed to the parameters of high flexibility, elasticity, environmental stability, high mobility of charge carriers, high ability to adsorb gas molecules, and the perfection of the crystal structure (Young’s modulus: ∼1–1.28 TPa, tensile strength: ∼100 GPa, specific strength: 48,000 kN m/kg, density: 1.3–1.4 g/cm^3^, electrical conductivity:105 S m^–1^, theoretical current density: 4 × 10^9^ A/cm^2^, photoluminescence (excitation) NIR – 1100–1800 nm (700–1050 nm), Raman: RBM = ∼100–200 cm^–1^, thermal conductivity: 0.1–6600 W m^–1^ K^–1^).^38–44^ Despite all of this, materials synthesized in modern laboratories for targeted ammonia sensing are still challenging to optimize. For instance, Muthukumaran et al.,^45^ fabricated Fe_2_O_3_/CNT-based resistive sensors by a hydrothermal method. The sensor responded to ammonia starting at a concentration of 250 ppm, where the sensitivity did not exceed 1.5 at 150 °C. Haridas et al.,^46^ synthesized α-Fe_2_O_3_ functionalized graphene sheets by the hydrothermal method. The sensor showed maximum response to ammonia at 250 °C, where a response value of 13.5% was recorded for a concentration of 10 ppm, assigning a response/recovery time of 152/648 s, respectively. Beniwal et al.,^47^ fabricated an iron oxide/polyaniline (Fe_2_O_3_/PANI) composite-based ammonia sensor by electrospinning of PANI nanofibers and drop casting a layer of Fe_2_O_3_ on the substrate. The sensor recorded a sensitivity of 1.99% for ammonia at extremely low concentrations (0.5 ppm), but the response and recovery times (950/250 s) still needed improvement. Thus, our main Fe_2_O_3_ gas-sensing material, acting as a platform for converting the presence of the gas phase into an electrical signal, cooperates with MWCNTs nanoislands as a means of increasing the effective surface area and the speed of the sensor, leading to a synergistic effect for the Fe_2_O_3_/MWCNTs composite structure.

In this work, a novel Fe_2_O_3_/MWCNTs material was obtained by the e-beam deposition method for high-performance ammonia sensing. The synthesized nanocomposite material used for chemoresistive sensors underwent various characterization studies, such as SEM, TEM, EDX, Raman, FTIR, and XPS. Moreover, excellent ammonia-sensing results were recorded at 200 °C, and NH_3_ detection mechanisms were discussed. Thus, the novelty of the work was reflected in the synthesis of the Fe_2_O_3_/MWCNTs material using a cheap, simple, and reproducible technological method and the demonstration of a highly efficient, selective, and fast sensor based on it that detected low concentrations of ammonia.

## Experimental Section

### Materials

Fe_2_O_3_ nanopowder (15–45 nm, purity of 99.9%) was purchased from Alfa Aesar (Haverhill, MA) for the preparation of the e-beam sputtering target. The functionalized MWCNTs were acquired by Nanoshel-UK Ltd. (Cheshire, U.K.) to saturate the sensor surface and improve the gas-sensing parameters. The platinum (Pt, purity of 99.95%) target was obtained by Goodfellow Ltd. (Shanghai, China) for coating the active surface of the sensor as catalytic nanoparticles. Pure NH_3_ solution (99.99%) was purchased from VWR Inc. (A.C.S., Pennsylvania) and aqueous solutions of ammonia of various concentrations were prepared online. As the sensor substrates, we used Multi-Sensor-Platforms manufactured by TESLA BLATNÁ (Blatná, Czech Republic). Temperature annealing was performed by using a software-controlled furnace (Nabertherm-HT04/16), Nabertherm GmbH, Lilienthal, Germany. The gas-sensitive film was obtained using a factory-designed e-beam deposition system (VUP-5M, Ukraine, Kyiv). Pt catalytic nanoparticles were deposited by the VTC-600-2HD DC/RF Dual-Head High Vacuum 2″ Magnetron Plasma System (Zhengzhou CY Scientific Co., Ltd., Chain). The morphological investigation of the Fe_2_O_3_/MWCNTs material was performed with the SEM microscope of Mira 3 LMH (Tescan). The thickness of the sensing film was measured by a Film Thickness Measurement Profiler D-300 (KLA-TENCOR, California). The TEM measurements were conducted by a Jeol 2200 FS (JEOL Ltd., Tokyo, Japan) instrument with 200 kV of accelerating voltage. The Electrochemical Workstation ZIVE SP1 (WonATech Co., Ltd., Seoul, Korea) and Keithley 4200-SCS Parameter Analyzer (Keithley/Tektronix Ltd., Ohio) techniques were used for sensor study by impedance spectroscopy.

### Preparation of the NH_3_ Sensor

To prepare a target for e-beam deposition, Fe_2_O_3_ nanopowder was preheated at 400 °C for 3 h to remove moisture absorbed from the atmosphere. The powder was then subjected to 10 h of thorough mixing and grinding, and then pressed into a tablet with 4 mm in length and 3 mm in width. The obtained target was subjected to a final heat treatment at 750 °C for 8 h, which provided it with mechanical strength. The target was placed inside a graphite anode in the e-beam sputtering chamber. Electrons from the tungsten cathode were accelerated by an electric field toward the anode, bombarding the Fe_2_O_3_ target with a high kinetic energy. Nanoparticles detached from the target were deposited in a vacuum environment and condensed onto the sensor substrate, forming a dense sensitive film (Figure 1). To improve the adhesion properties of the film and substrate and more efficiently grow the film consisting of nanograins, the substrate was kept at 250 °C during the deposition process. The distance between the substrate and target was chosen to be 7 cm, which resulted in a more homogeneous film.

**Figure 1:**
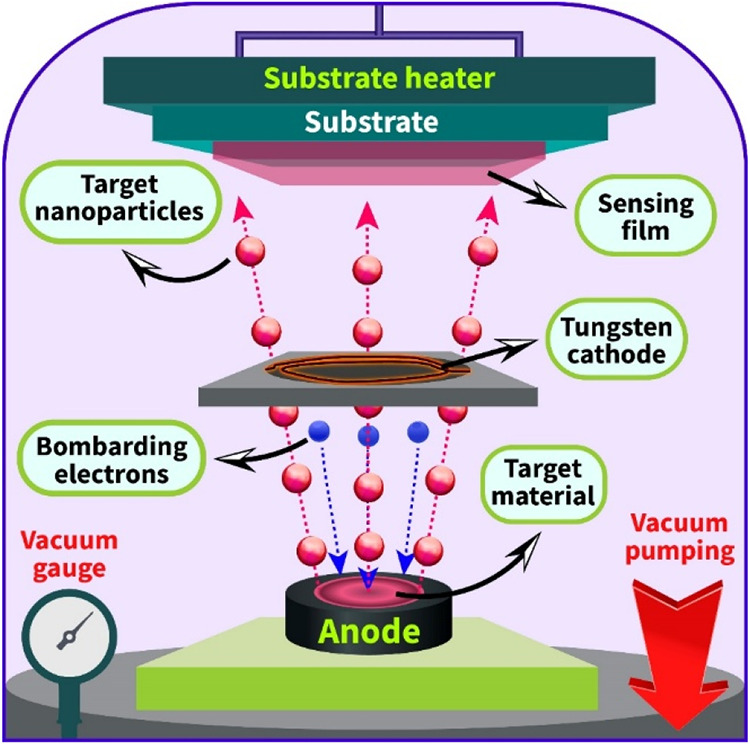
Schematic representation of the film growth process by the e-beam deposition system.

After obtaining the Fe_2_O_3_ nanofilm, a 4 cm long and 3 cm wide tablet was preprepared from the MWCNTs powder as an e-beam deposition target. MWCNTs-based nanoclusters were deposited from the obtained target onto the surface of the gas-sensitive (Fe_2_O_3_) film. The surface of the nanostructure with a morphology favorable for the adsorption of gas molecules was finally saturated with platinum (Pt) nanoparticles, which act as catalytic islands, contributing to both an increase in the sensor’s operating speed and its response.^48,49^ Pt catalytic nanoparticles are quite effective for the detection of ammonium, as they promote both the rapid dissociation of the ammonium molecule and its subsequent reactions with surface oxygen species.^50,51^ All technological regimes for the preparation of the NH_3_ sensor are summarized in Table 1 and illustrated in Figure 2a.

**Figure 2:**
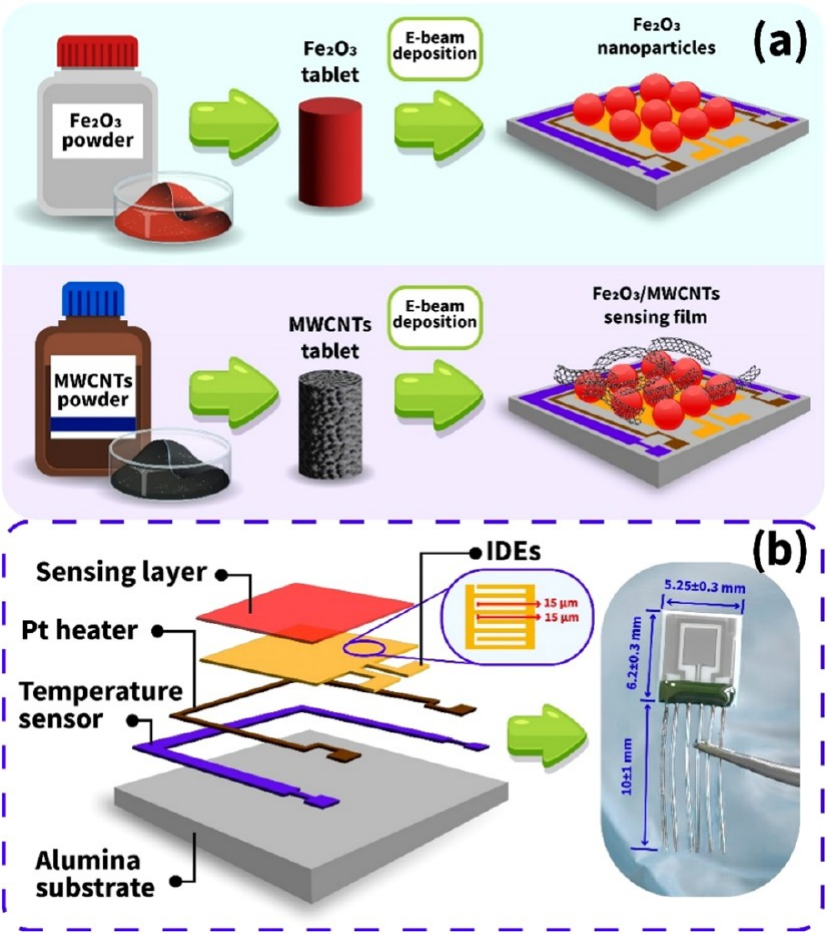
Schematic block diagram of the NH_3_ sensor fabrication (a), schematic illustration, and actual photograph of the sensor (b).

**Table 1:** Technological Modes for Obtaining the NH_3_ Sensor

process	sputtering duration	working pressure [Pa]	sputtering gas	substrate temperature [°C]	cathode current [mA]	anode voltage [kV]	base pressure [Pa]
E-beam deposition (Fe_2_O_3_ nanograins)	25 min	9 × 10^–2^		250		0.5	5 × 10^–3^
E-beam deposition (MWCNTs)	4 min	8 × 10^–2^		250		0.5	6 × 10^–3^
DC magnetron sputtering (Pt catalytic particles)	4 s	6 × 10^–2^	Ar	250	200		1 × 10^–3^

We used factory-designed alumina platforms as an effective substrate for sensor applications. The platinum electrodes, temperature sensor, and heater were integrated onto the same platform (Figure 2b). Effective recording of the change in resistance of the gas-sensitive film was carried out through interdigitated ohmic electrodes (IDEs), and the spiral heater made it possible to keep the sensor at a temperature of up to 350 °C.

### Gas Sensor Measurements

The gas-sensing characteristics of the sensor were thoroughly investigated by using the GST PRO gas sensor testing system. A specially designed 1 L chamber housed the necessary attributes for measuring NH_3_ and recording other environmental parameters (Figure 3). Such a relatively large volume of the measurement chamber makes it possible to obtain lower concentrations inside and to measure (attach) not one but up to six sensor structures. A special heater was installed at the bottom of the chamber to raise the sensor temperature from room temperature to 500 °C. The humidity level and air temperature in the chamber were dynamically controlled by using specific sensors. A mechanical pump providing a low vacuum was connected to the chamber for measurements in an oxygen-free environment. The system was equipped with high-precision mass flow controllers (MFCs) to keep the necessary gas concentration in the chamber for substances in the gas phase. In the case of ammonia, we used the method of evaporation from the liquid phase to the vapor one. A special crucible was prepared in advance in the chamber, the heater of which brought the temperature to 200 °C. A calculated amount of ammonia aqueous solution was introduced there, which, at high temperature, immediately converted to the gaseous phase. Knowing the volume of the chamber, the temperature and pressure inside the chamber, and the number of moles of ammonia in the evaporated solution, the ammonia concentration in the chamber was calculated.^21^ To change the concentration, the relative concentration of the ammonia aqueous solution was changed. For the DC power supply and data recording, a power source (Twintex TP-2303) and a digital multimeter (Keithley, DMM 7510) were externally connected to the chamber. Here, a special system for evaporating liquids was also made of the factory, which was capable of heating up to 200 °C.

**Figure 3:**
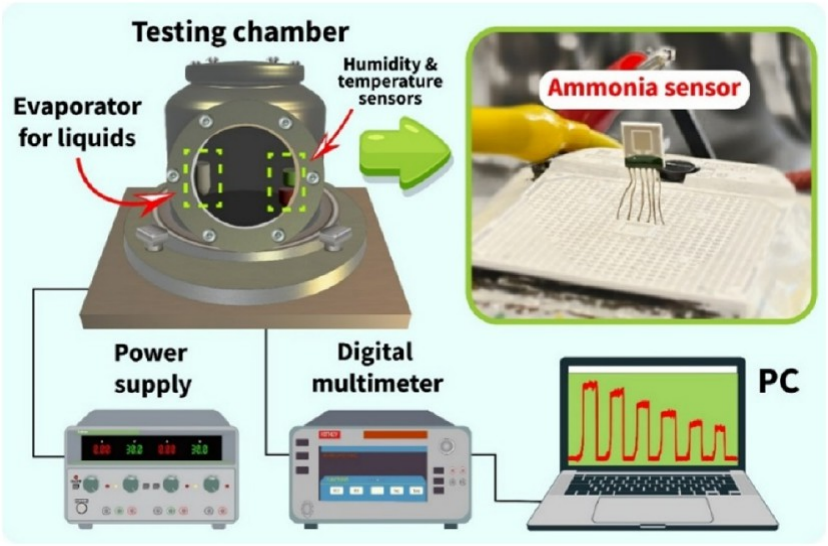
Schematic block diagram of the GST PRO gas sensor testing system.

The NH_3_ gas response was defined as a ratio of the sensor resistances in air and gas environment, respectively.

## Results and Discussion

### Characterization

Fe_2_O_3_ powder was subjected to characterization before pressing, where the morphology and size of the powder grains were of great importance. The nanopowder had a rather heterogeneous morphology before processing, with no particular grain homogeneity observed (Figure 4a). In contrast, a more porous system was formed after pressing and finally annealing the powder. The solid target formed here consisted of rather large grains or pores (10–25 μm) (Figure 4b), which did not significantly affect the sputtering parameters. A high-energy electron beam is believed to break surface bonds, especially when the surface morphology is porous, which is a favorable condition for high deposition rates. In the case of targets with harder and stronger bonds, it is possible to observe melting of the target material, and the sputtering can be transformed into thermal evaporation. If we use oxides as the target material, this can lead to the transformation of the oxide to a metal, since during the phase change, the oxygen species can be completely sucked out by the vacuum environment.^52^

**Figure 4:**
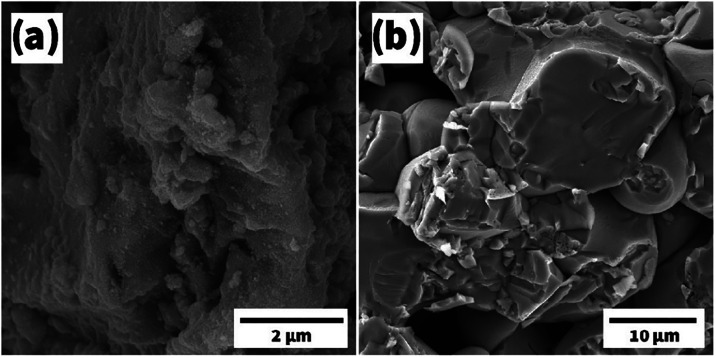
SEM images of the powder (a) and target (b) for the Fe_2_O_3_ material.

In electron beam sputtering, a beam of electrons emitted from a cathode is accelerated by an electric field and directed toward the target material with high energy. Depending on the number of these electrons (the brightness of the cathode) and the magnitude of the applied electric field, the particles detached from the target can have different sizes.^53^ In the case of our chosen technological regimes (see Experimental Section), the SEM images of the surface of the Fe_2_O_3_ film suggest a granular structure with high homogeneity (Figure 5a,b). As a gas-sensitive film, obtaining a surface with a granular/porous morphology is important, where increasing the effective surface area significantly contributes to improving the adsorption capacity of gas molecules. Here, a grain size range of 20–25 nm was recorded (Figure 5c), which is comparable to twice the Debye length of the Fe_2_O_3_ material (12.3 nm),^54^ representing the most favorable prerequisite for obtaining high sensitivity.^55^

**Figure 5:**
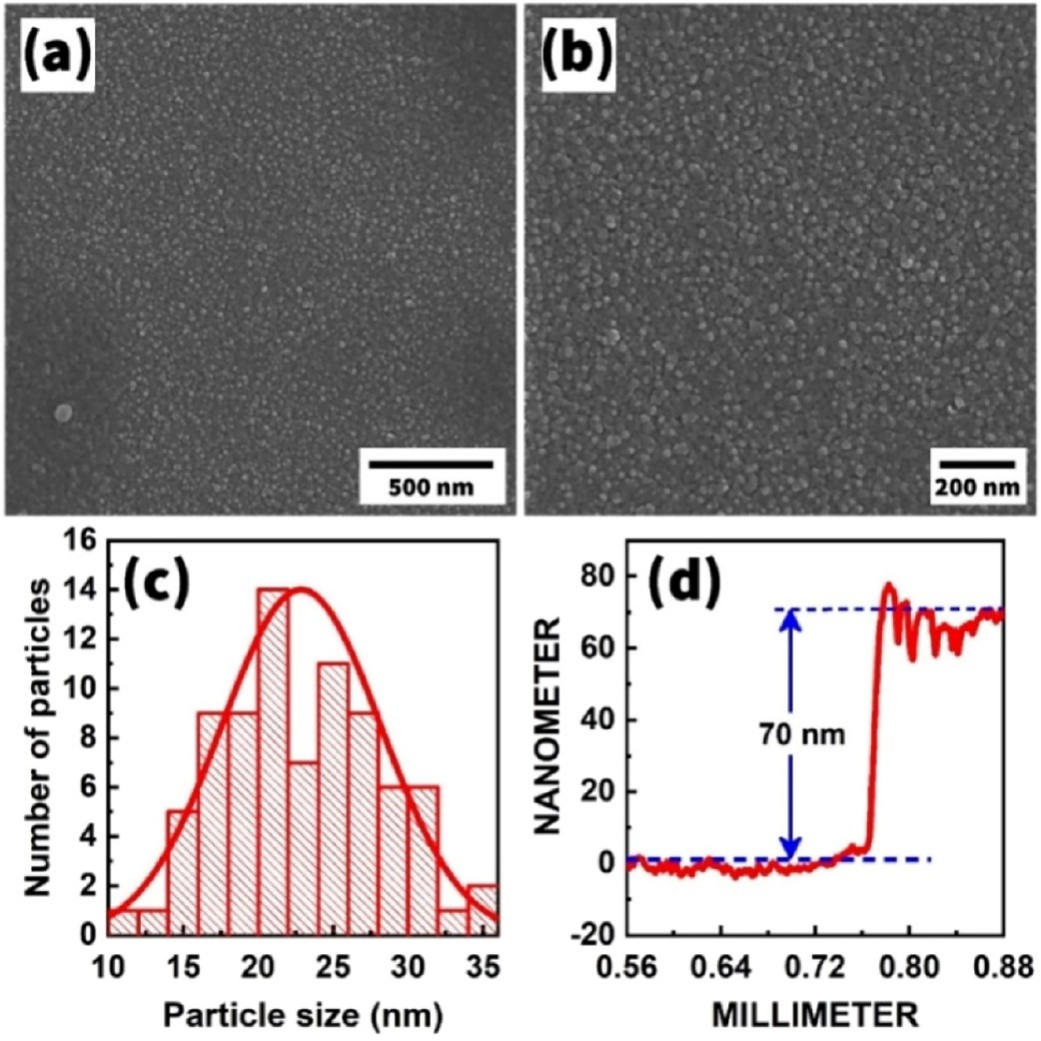
SEM images (a, b), particle size histogram (c), and thickness measurement results (d) of the Fe_2_O_3_ film.

A significant indicator of improving gas-sensing parameters is the thickness values of the active film, which can be adjusted depending on the gas type and operating temperature. Typically, in the case of oxidizing gases, the use of thinner films (<40 nm) is advisible, since their molecules mainly interact with the semiconductor surface, penetrating to a maximum depth of 10–15 nm.^56^ In this case, using a thicker film becomes impractical, leading to a decrease in sensitivity due to a reduced fraction of the depleted layer in the total film thickness. For gases with lower atomic radius and reducing characteristics, the use of thicker films (>40 nm) is advantageous, due to the penetration of molecules into deeper layers of the film and therefore increased sensitivity.^57^ Thus, we estimated the thickness of the obtained Fe_2_O_3_ films, which floated around 70 nm (Figure 5d). The use of gas-sensitive films with similar thicknesses for ammonia as the reducing gas has led to positive results in terms of optimizing gas-sensing parameters,^58^ which was also confirmed in this work. To reveal the crystal structure of the Fe_2_O_3_ film, it was subjected to a TEM study. Here, the material’s grain structure was also visible (Figure 6a,b), the dimensions of which (20–30) were in good agreement with the SEM results.

**Figure 6:**
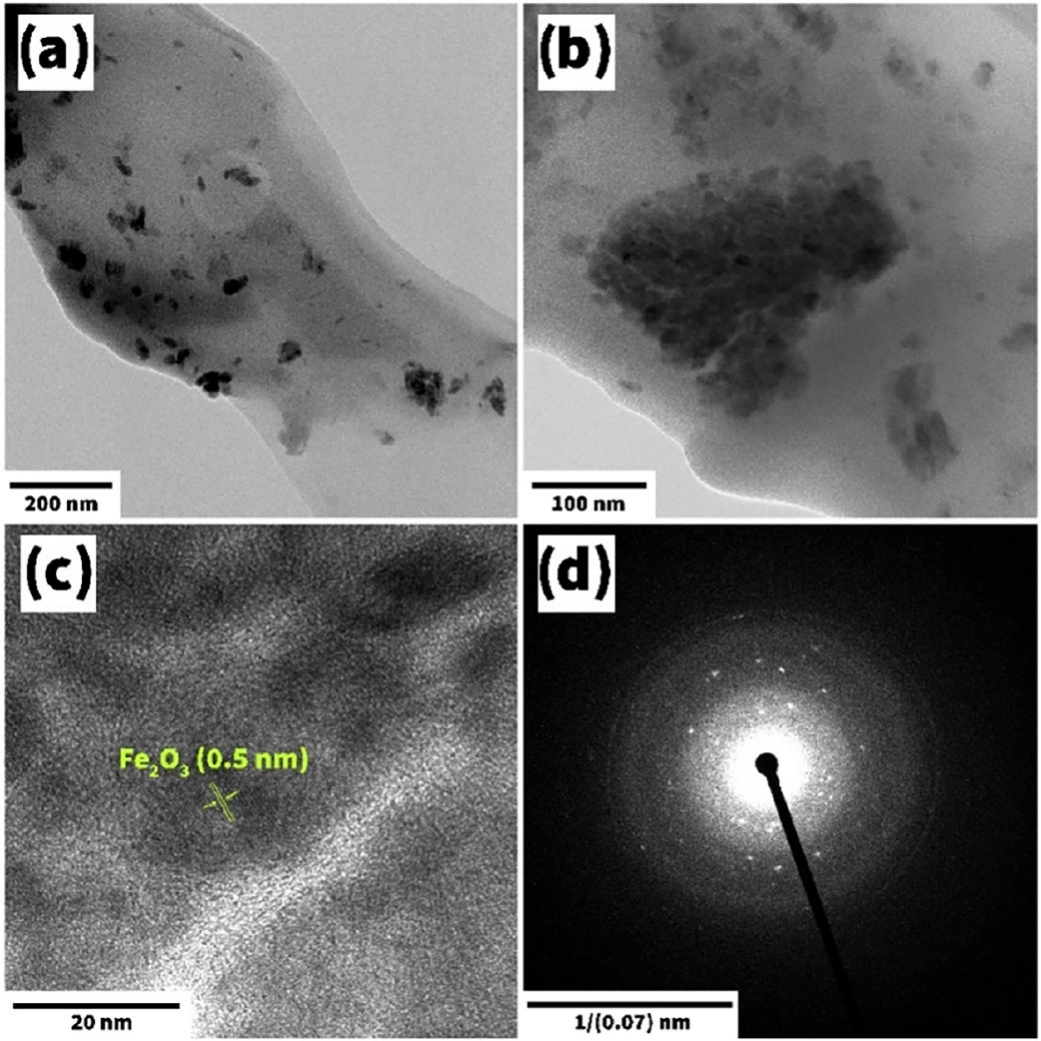
Low (a, b) and high-resolution (c) TEM images and the SAED pattern (d) for the Fe_2_O_3_ material.

Figure 6c represents high-resolution transmission electron microscopy (HRTEM) diffraction of the α-Fe_2_O_3_ material. The interplanar spacing for this material was indexed to be 0.5 nm, representing the lattice fringes of Fe_2_O_3_ nanoparticles with the plane of hexagonal α-Fe_2_O_3_.^59^ The selected area electron diffraction (SAED) pattern for the Fe_2_O_3_ material (Figure 6d) confirmed the polycrystalline structure of the film.^21^

The cotton-like mass of the MWCNTs and its target for e-beam sputtering were subjected to an SEM study, revealing the appearance and size of the nanotubes presented here. In the SEM image at lower magnification (Figure 7a), nanotubes with a diameter of 15–30 μm and a length of several hundred μm were evident, which did not have an ordered arrangement. Evidently, large tubes were made up of many smaller nanotubes that were embedded in each other in a network-like and irregular manner with diameters ranging from 80 to 200 nm (Figure 7b).

**Figure 7:**
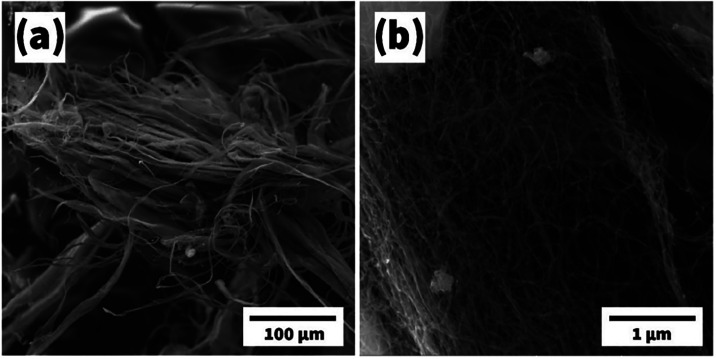
SEM images for MWCNTs with scale bars of 100 μm (a) and 1 μm (b).

Furthermore, TEM and HRTEM were also applied to characterize attentively the morphology and crystalline properties of the MWCNTs. There, a cylindrical shape of a nanotube with a diameter of about 200 nm was clearly visible (Figure 8a,b), where local breaks in the crystal lattice were also noticeable. The characteristic interplanar spacing of MWCNTs was observed to be 0.34 nm (Figure 8c,d), revealing its hexagonal tubular nature.^60^

**Figure 8:**
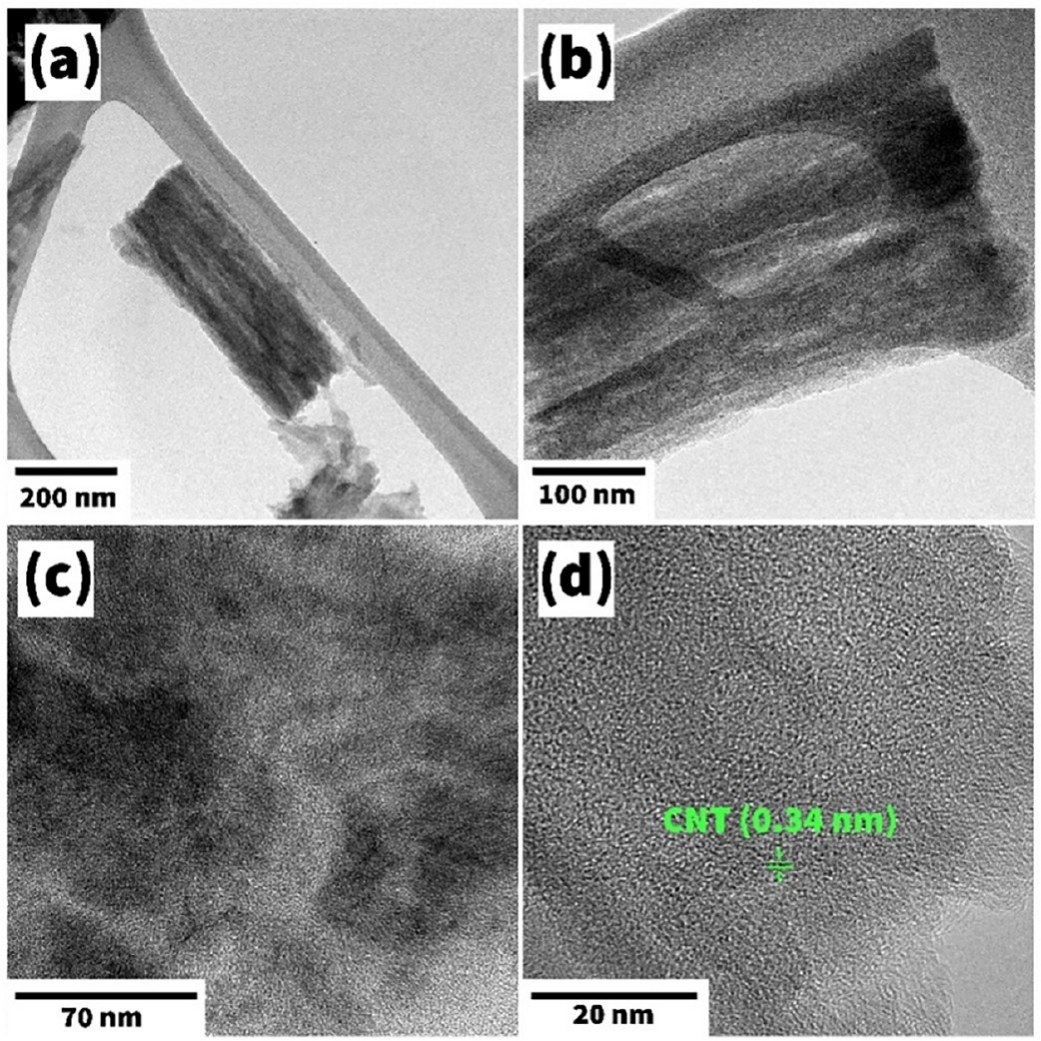
Low (a, b) and high (c, d) resolution TEM images for the MWCNTs.

Besides, the hexagonal structure of the MWCNTs was also expressed in the Raman spectroscopy, representing bands of D (defect) and G (graphite band) at 1351 and 1552 cm^–1^, respectively (Figure 9). It indicated the structural defect of the MWCNTs,^61^ being in good agreement with the TEM measurements. The spectrum due to the silicon substrate showed a well-pronounced peak at 500 cm^–1^.^62^

**Figure 9:**
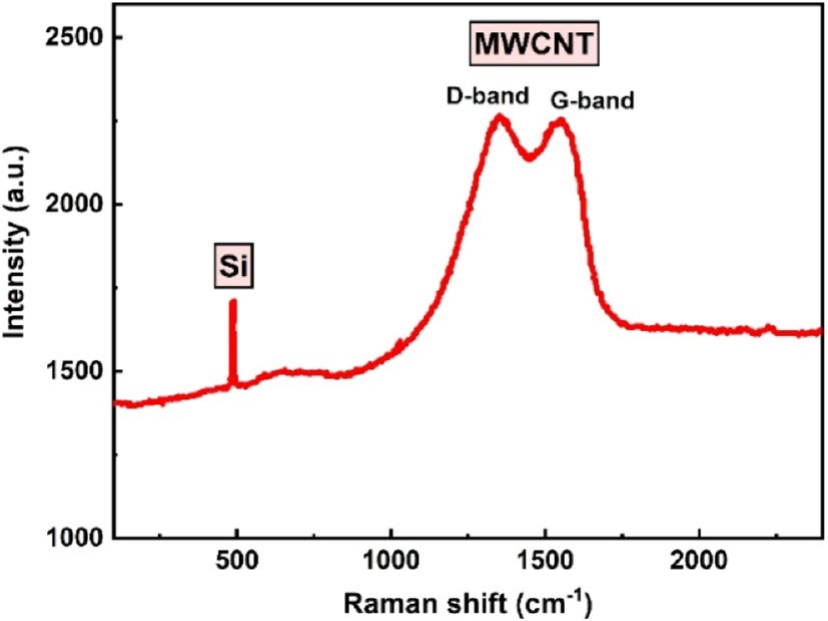
Raman spectra of the MWCNTs.

To estimate the actual concentrations of elements available in a gas-sensitive material, the elementary EDX analysis was applied to the Fe_2_O_3_/MWCNTs structure (Quantax 200 with XFlash 6|10 detector (Bruker) with a resolution of 127 eV and 15 kV of accelerating voltage). The chemical elements of iron (Fe), oxygen (O), and carbon (C) present in this structure were expressed in the EDX spectrum with pronounced peaks (Figure 10). The significant concentration of oxygen (20.34 atom %) here is due to both the iron oxide’s native oxygen species and surface-localized oxygen centers absorbed from the atmosphere. The low carbon concentration (5.04 atom %) is due to the short deposition time (4 min) for the e-beam deposition method, resulting from sparsely distributed MWCNT islands on the Fe_2_O_3_ surface.

**Figure 10:**
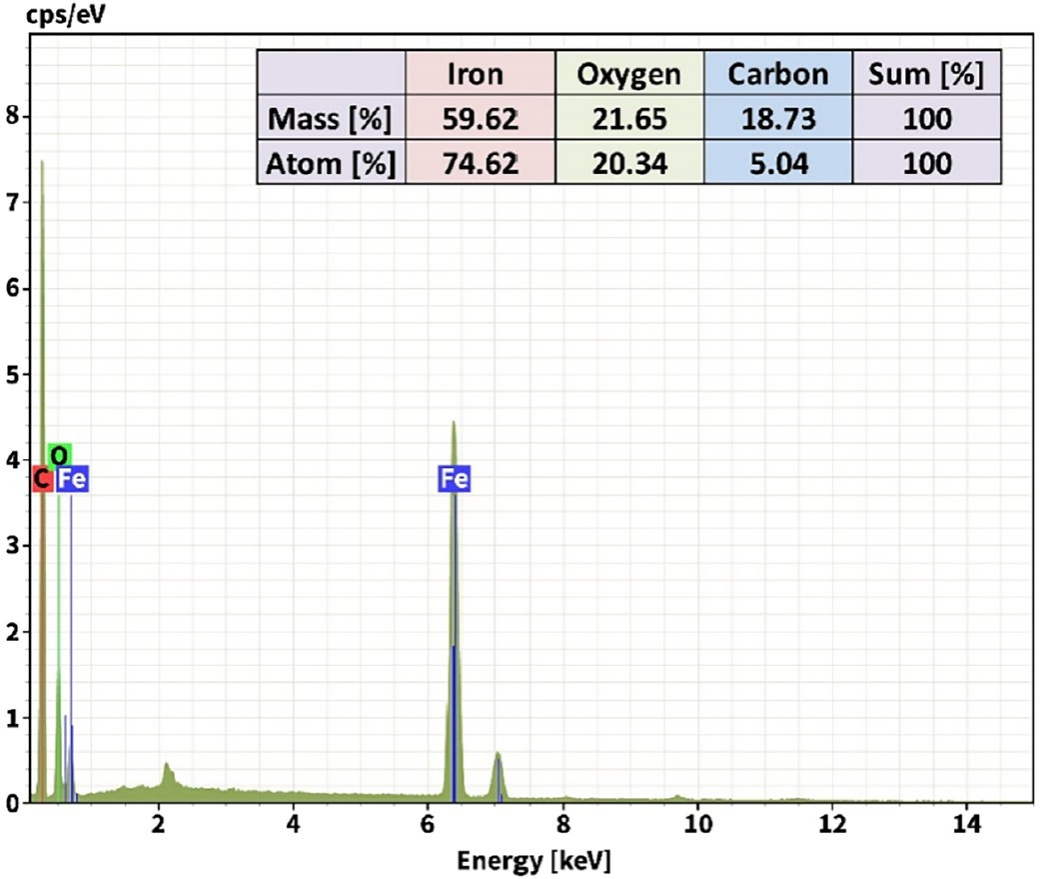
EDX spectrum of the Fe_2_O_3_/MWCNTs material.

FTIR spectra of the Fe_2_O_3_/MWCNTs and Fe_2_O_3_ materials were extracted in the range of 400–4000 cm^–1^ (Figure 11). In the spectrum of pristine Fe_2_O_3_, two peaks appeared, corresponding to the values of 529 and 473 cm^–1^. These were characterized by the Fe–O bond due to the vibrational mode.^63^ Besides, in the FTIR spectrum of the Fe_2_O_3_/MWCNTs material, peaks were newly revealed in the range of 1628–1106 cm^–1^, corresponding to C–C and C=O stretches.^64^ The addition of MWCNTs to the surface of Fe_2_O_3_ led to the formation of a composite material with the modified hematite surface, causing band shifting in the FTIR spectrum from 529 to 674 cm^–1^. Moreover, the intensity peak at 2920 cm^–1^ was associated with the C–H stretching vibrations in the Fe_2_O_3_/MWCNTs material.^65^

**Figure 11:**
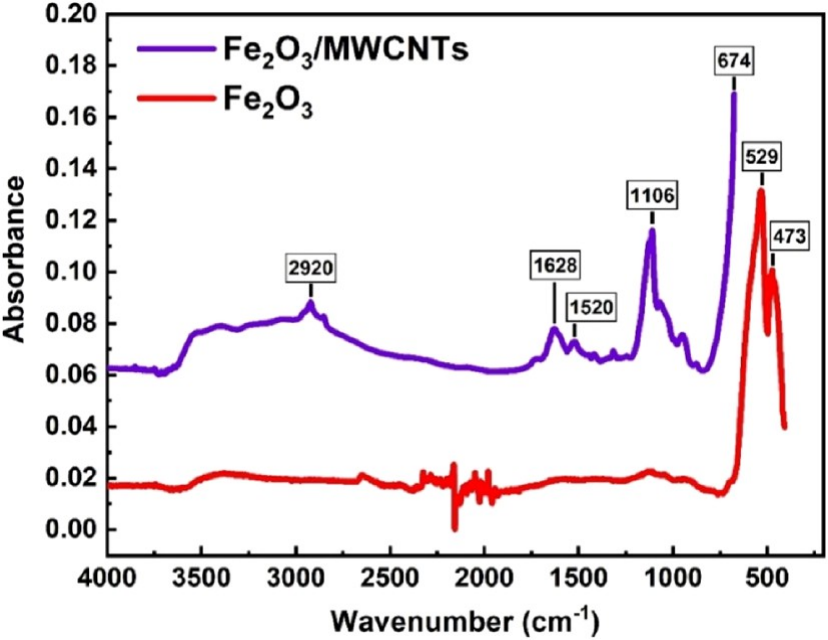
FTIR spectra of the Fe_2_O_3_/MWCNTs and Fe_2_O_3_ materials.

The Fe_2_O_3_/MWCNTs structure was subjected to XPS studies to reveal the types of bonds between elements present in the material and to identify their nature. The XPS survey spectra of the Fe_2_O_3_/MWCNTs material revealed three pronounced peaks corresponding to the characteristic bonds of Fe 2p, O 1s, and C 1s (Figure 12a). The high-resolution XPS spectrum of Fe 2p represented the main intensity peaks of Fe 2p_3/2_ and Fe 2p_1/2_ corresponding to 724 and 711 eV, respectively (Figure 12b). The feature peaks of Fe_2_O_3_ were also visible as the satellite peaks with their high binding-energy side.^66^ The high-resolution XPS spectrum of O 1s revealed a main peak at 529 eV representing lattice oxygen atoms of Fe_2_O_3_ (Figure 12c). The less pronounced peak at 531 eV was due to either OH groups or the absorbed water molecules on the sensing surface.^67^ Moreover, the peaks of C–C and C­(O)C bonds were available in the C 1s spectrum at 284 and 289 eV, respectively (Figure 12d). These confirmed the presence of the graphitic nature of carbon nanotubes in the material.^66^

**Figure 12:**
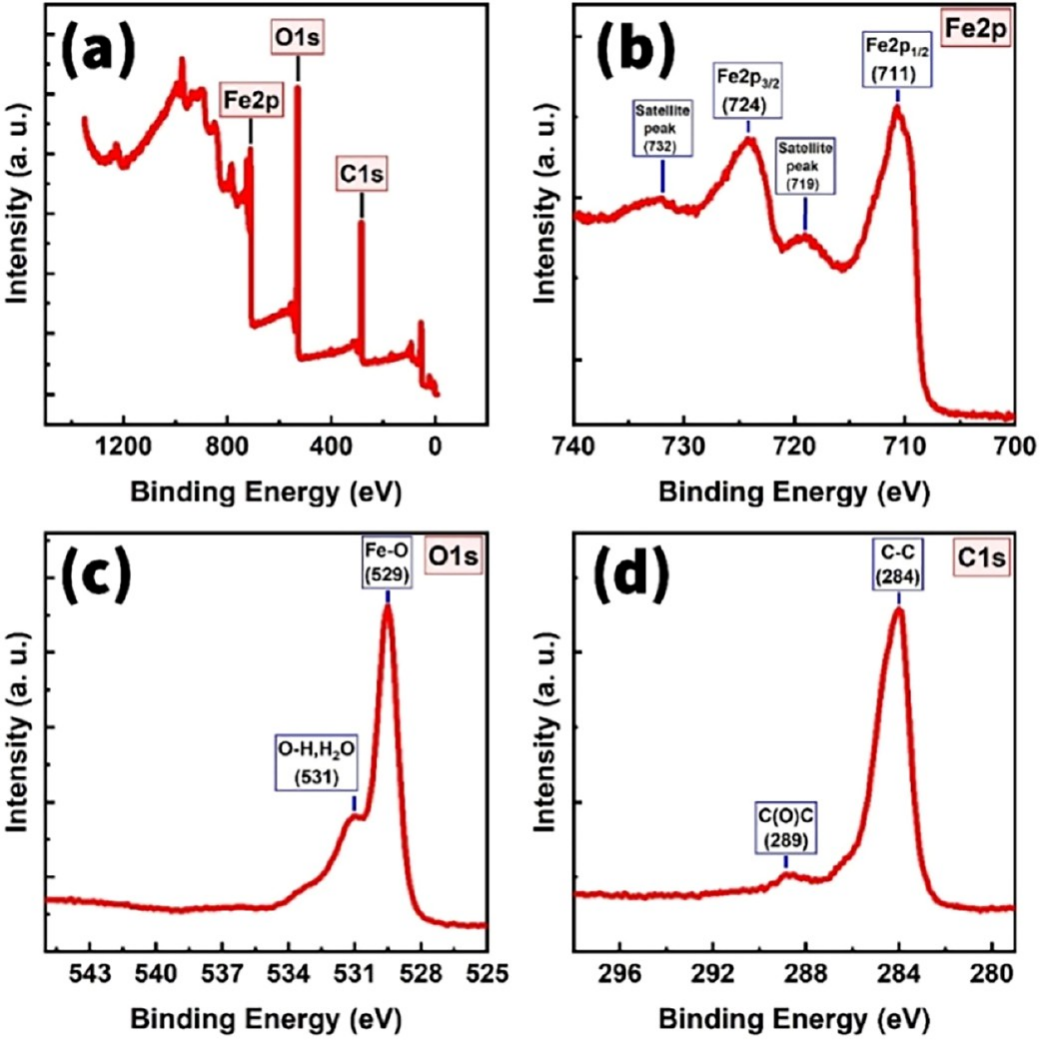
XPS survey spectrum (a), high-resolution XPS spectrum of Fe 2p (b), C 1s (c), and O 1s (d) for the Fe_2_O_3_/MWCNTs material.

### NH_3_ Sensing Performance

Typically, the temperature-dependent behavior of thin films is characterized by a high sensitivity of the surface resistance to ambient atmospheric conditions. At lower temperatures (<100 °C), metal oxide semiconductors are usually quite stable with respect to temperature due to their high band gap energy values and the lack of chemisorption of oxygen species. At higher temperatures (>100 °C), thermal excitation in a semiconductor generates free charge carriers that participate in the electrical conduction process.^68^ The Fe_2_O_3_/MWCNTs film exhibited extremely high resistance (15–25 GΩ) from room temperature to 100 °C, and then a monotonic decrease in resistance up to 300 °C was observed due to thermally activated charge carriers (Figure 13a). When the film was cooled in the opposite direction, the resistance behavior was almost mirror-imaged. It is assumed that the effect of oxygen species adsorption along the temperature sweep was quite weak since no resistance peaking was observed due to the utilization of free charge carriers by adsorption processes. Thus, the sensor response values to 250 ppm ammonia were observed at temperatures corresponding to the measurable resistance range to determine the most effective operating temperature of the sensor. The sensor exhibited a pronounced maximum peak at 200 °C, where the sensor resistance changed more than 12.2 times (Figure 13b). It is noteworthy that already at 300 °C, the sensor almost completely lost its response due to the nearly impossible adsorption of ammonia gas molecules on the hot surface. At the most favorable temperature for ammonia detection (200 °C), the sensor responses were measured in the concentration range of 3–650 ppm. The low detection limit of the sensor (3 ppm) corresponded to a response value of 2.7, which reached 12.2 at a concentration of 250 ppm of NH_3_ (Figure 13c). The seven measured response curves corresponding to different concentrations had well-reproducible and consistent appearances. In the range of 3–650 ppm of NH_3_ concentrations, a linearity of the sensor response was not observed, but rather saturation behavior was exhibited (Figure 13d). Sometimes this tendency to saturation of the response/concentration dependence is characteristic of chemisorption sensors, where the molecular blockade of the majority of the active surface may be the main reason for this.^69^

**Figure 13:**
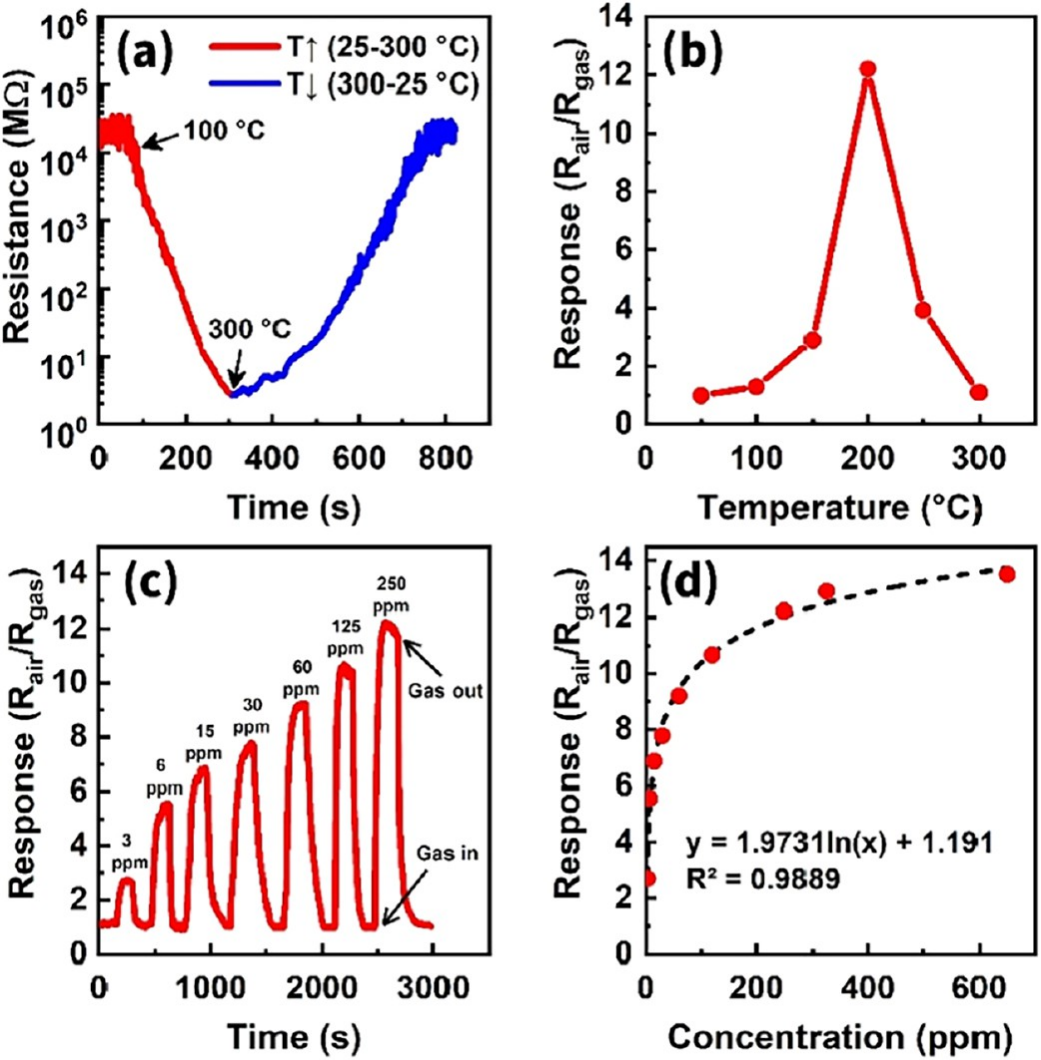
(a) Sensor resistance vs operating temperature, (b) sensor response vs operating temperature at 250 ppm of NH_3_, (c) dynamic response curves of the sensor to different concentrations of ammonia at 200 °C, (d) sensor response vs NH_3_ concentration at 200 °C.

The important indicators of sensor performance, such as response and recovery times, were evaluated by assigning them to 40 and 25 s, respectively (Figure 14a). The sensor was particularly notable for its extremely short recovery time, as the desorption of gas molecules in chemisorption sensors usually takes a long time.^70^ It is believed that the adsorbed ammonia molecules interacted specifically with the film surface and did not penetrate into the interior parts of the carbon nanotubes and Fe_2_O_3_ nanograins. Moreover, as a significant parameter for the sensing performance, the sensor reproducibility was evaluated, representing six reversible cycles of the sensor toward 30 ppm ammonia at 200 °C (Figure 14b). The excellent reproducibility of the sensor, almost free of deviations from the initial response value, was attributed to the parametric stabilization of basic metal oxide (Fe_2_O_3_) combined with carbon nanotubes.

**Figure 14:**
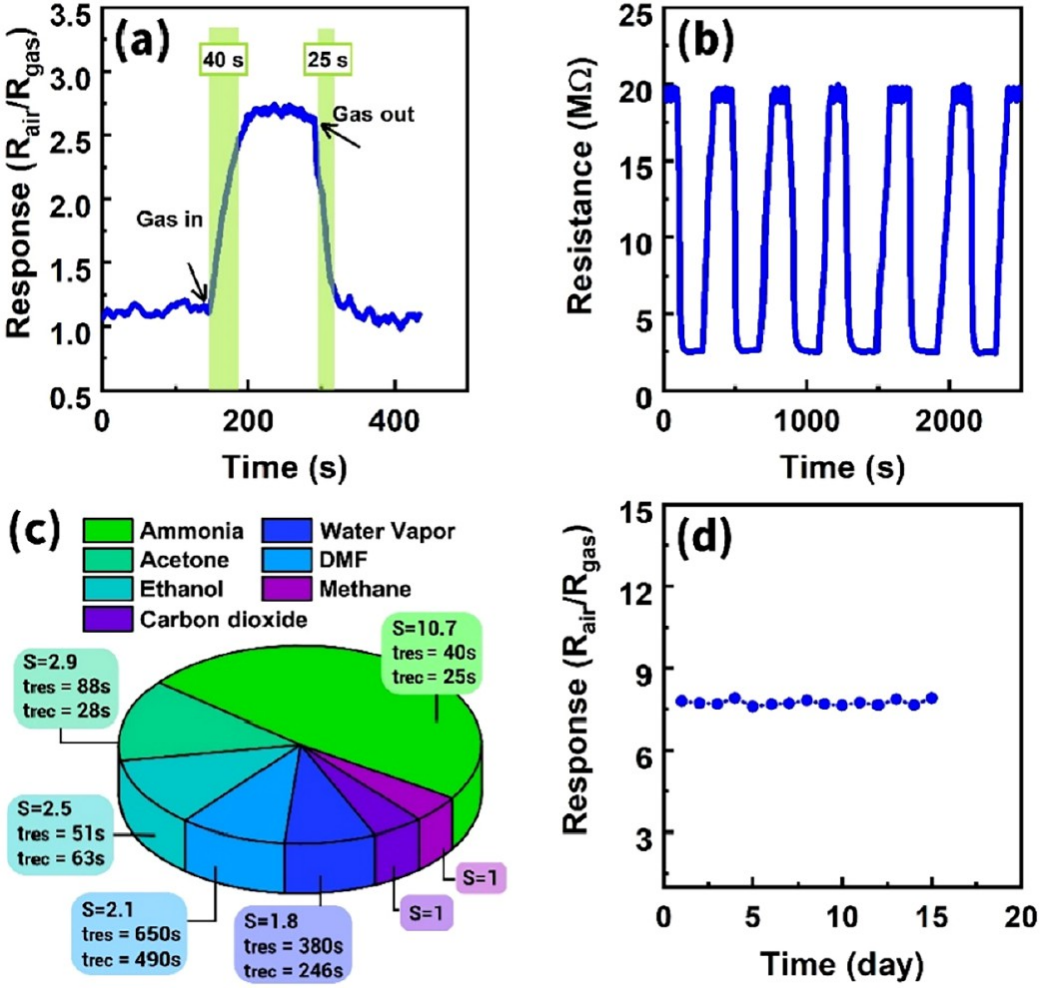
(a) Real-time response curve of the sensor at 3 ppm ammonia, representing the response and recovery times at 200 °C, (b) repeatability tests of the sensor to 30 ppm ammonia at 200 °C, (c) selectivity of the sensor for NH_3_ at 200 °C, and (d) long-term stability of the sensor for ammonia at 200 °C.

Furthermore, the selectivity tests were carried out toward 120 ppm of NH_3_ (*S* = 10.7) and other trace gases such as 120 ppm acetone, 120 ppm ethanol, 5000 ppm carbon dioxide, 3000 ppm water vapor, 120 ppm dimethylformamide (DMF), and 5000 ppm methane representing response values of 2.9, 2.5, 1, 1.8, 2.1, and 1, respectively (Figure 14c). The sensor’s markedly higher sensitivity to ammonia at lower concentration compared to those of interfering gases demonstrated its highly selective displayment. Additionally, the long-term stability of the response for the Fe_2_O_3_/MWCNTs sensor was estimated toward 30 ppm ammonia (*S* = 7.8) at 200 °C (Figure 14d). The sensor response was measured for 15 consecutive days under the same physical conditions; it did not significantly deviate from the initial value, but slight fluctuations were observed, proving the high temporal stability of the sensor.

The Fe_2_O_3_/MWCNTs sensor was also studied by impedance spectroscopy, extracting the Nyquist curves in air and the gas environment. The concentration of 325 ppm ammonia significantly shifted the semicircular Nyquist curve of the sensor toward a lower resistance range (Figure 15a). Using the impedance studies in the frequency range of 1–100 Hz, we proposed an equivalent electrical circuit of the Fe_2_O_3_/MWCNTs.^71^ The equivalent circuit consisted of a series resistor (*R*_s_ = 564.6 Ω) and two RC circuits connected to it (Figure 15b). Presumably, the first *R*_1_ *C*_1_ (*R*_1_ = 3.9 MΩ, *C*_1_ = 32.5 pF) circuit can be attributed to the junction of Fe_2_O_3_ grains, while the second *R*_2_ *C*_2_ (*R*_2_ = 88.1 kΩ, *C*_2_ = 66.4 nF) circuit was responsible for the MWCNTs islands.

**Figure 15:**
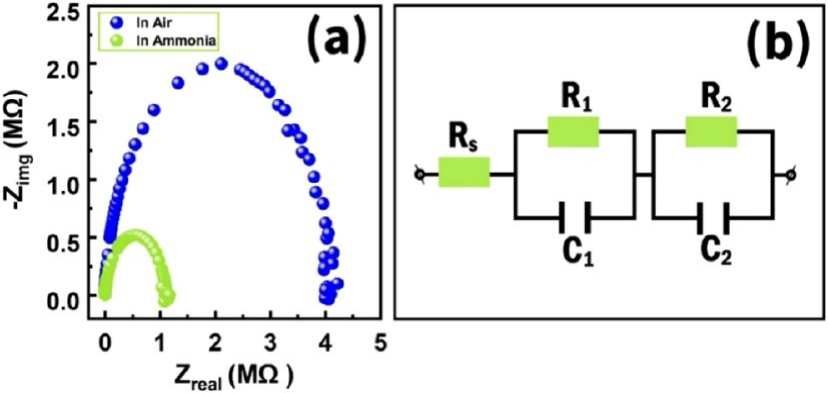
(a) Sensor Nyquist curves in air and at 325 ppm of NH_3_ (b), and the proposed equivalent electrical circuit of the Fe_2_O_3_/MWCNTs sensor.

The performance characteristics of our sensor are given in comparison with the characteristics of other existing sensors, highlighting the relevance of this work. The gas-sensing parameters of the Fe_2_O_3_/MWCNTs sensor, such as operating temperature, response, and detectable concentrations of NH_3_, were clearly comparable to the characteristics of the existing sensors (Table 2).

**Table 2:** Literature Comparison of the NH_3_ Sensing Properties of Various Chemoresistive Sensors to Those of Our Fe_2_O_3_/MWCNTs Sensor

sensing materials	operating temperature [°C]	ammonia concentration [ppm]	response [*R*_air_/*R*_gas_]	references
NiO thin film	150	1000	316%	^72^
Pt/ZnO porous film	330	100	324	^73^
In_2_O_3_/Co_3_O_4_ flower-like nanocomposites	250	10	7.5	^74^
h-MoO_3_ nanorods	200	10	46%	^75^
MWCNTs/PANI	25	33	2	^76^
PANI/CuFe_2_O_4_ heterostructure	25	5	27.37%	^77^
ZnO nanoflakes	250	3	80%	^78^
(Al)-doped ZnO thin film	100	25	12	^79^
Fe_2_O_3_/MWCNTs nanocomposites	150	250	3	this work
Fe_2_O_3_/MWCNTs nanocomposites	200	250	12.2	this work
Fe_2_O_3_/MWCNTs nanocomposites	200	3	2.7	this work

### Sensing Mechanism

Physicochemical processes occurring on the sensor’s active surface are the main cause of the change in resistance of chemoresistive sensors due to the presence of the target gas. The adsorption of gaseous species from the environment on the surface of semiconductor materials is a well-known phenomenon, which is the main reason for the chemiresistive interaction.^80^ Due to the sufficient amount of oxygen species in the atmosphere and their nonpassive chemical nature, they are usually adsorbed on the surface of solid materials and interact with the lattice. Oxygen species adsorb on the surface of a metal oxide under atmospheric conditions and form localized ions through electron exchange (eqs 1–4). Thus, the surface of the semiconductor becomes depleted of electrons, and space charge layers are formed around the nanograins.^81,82^

1 $$O_{\left(\mathrm{gas}\right)}↔O_{\left(\mathrm{ads}\right)}$$

2 $$O_{2\left(\mathrm{gas}\right)}↔O_{2\left(\mathrm{ads}\right)}$$

3 $$O_{2\left(\mathrm{ads}\right)}+e^{-}\to {O_{2\left(\mathrm{ads}\right)}}^{-}$$

4 $$O_{\left(\mathrm{ads}\right)}+e^{-}\to {O^{-}}_{\left(\mathrm{ads}\right)}$$

Ammonia in the gas phase is adsorbed on the film surface and reacts with prelocalized oxygen ions. Thus, water and nitric oxide molecules are produced as a result of the following assumed chemical reactions^78,83,84^

5 $${\mathrm{NH}}_{3\left(\mathrm{gas}\right)}\to {\mathrm{NH}}_{3\left(\mathrm{ads}\right)}$$

6 $$4{\mathrm{NH}}_{3\left(\mathrm{ads}\right)}+5{O_{2\left(\mathrm{ads}\right)}}^{-}\to 6H_{2}O+4\mathrm{NO}+5e^{-}$$

The reaction products also contain excess electrons, which fall back into the conduction band of the Fe_2_O_3_ material, reducing its resistance (Figure 16). This is typical behavior of a reducing gas (ammonia) in an n-type Fe_2_O_3_ semiconductor, the change in the resistance of which is expressed by the sensor response.

**Figure 16:**
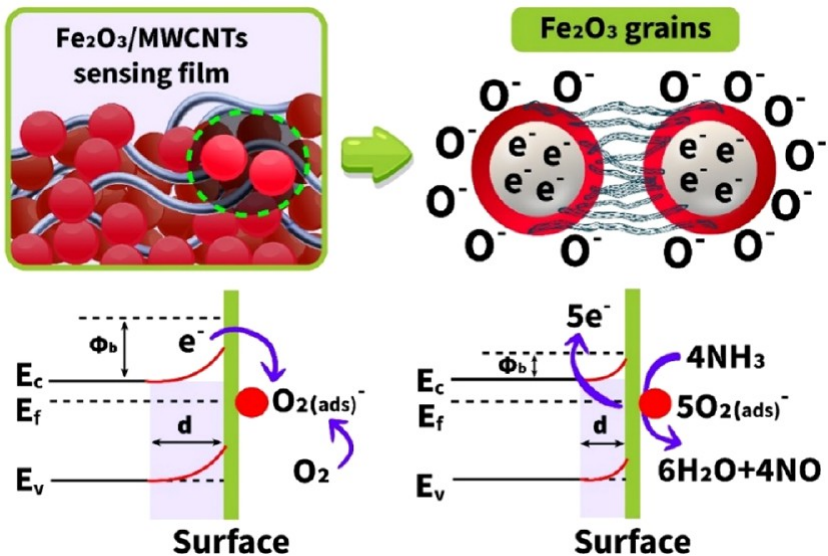
Ammonia-sensing mechanism for the Fe_2_O_3_/MWCNTs material.

Here, MWCNTs isolated on the Fe_2_O_3_ film surface play an important and indispensable role in the entire sensing mechanism of ammonia gas. It is believed that these are unique electrical channels between the neighboring Fe_2_O_3_ nanograins. Having an incomparably high mobility of charge carriers,^37^ this leads to a dramatic improvement in the speed of the sensor. Besides, the large effective surface area of the nanotubes also dramatically increases that of the Fe_2_O_3_/MWCNTs material, increasing its ability to adsorb more gas molecules. We have proven this claim using the Brunauer–Emmett–Teller (BET) theory with the content presented below. One of the methods to determine surface area and porosity characteristics is the BET theory based on the extension of the Langmuir theory, which originally described monolayer adsorption. In contrast to the Langmuir model, which posits a single adsorbed layer, BET theory accounts for multilayer adsorption.^85^ Thus, the BET equation is expressed as

7 $$\frac{p/p_{0}}{v_{a}\left(1-p/p_{0}\right)}=\frac{c-1}{v_{m}c}+\frac{1}{v_{m}c}$$

where *p* is the equilibrium pressure, *p*__0__ is the saturation pressure of the adsorbates at the measurement temperature, *v*_a_ is the adsorbed gas volume, *v*_m_ is the volume of the monolayer adsorbed gas, and *c* is the BET constant. Experimentally obtained pressure data during the gas response measurement were used to plot 1/[*v*_a_(1 – *p*/*p*_0_)] versus *p*/*p*_0_ (Figure 17a). This yielded a curve (Figure 17b) whose slope (*A*) and intercept (*I*) can be used to determine *v*_m_ and *c* using the following formulas^86^

8 $$c=1+\frac{A}{I}$$

9 $$v_{m}=\frac{1}{A+I}$$

**Figure 17:**
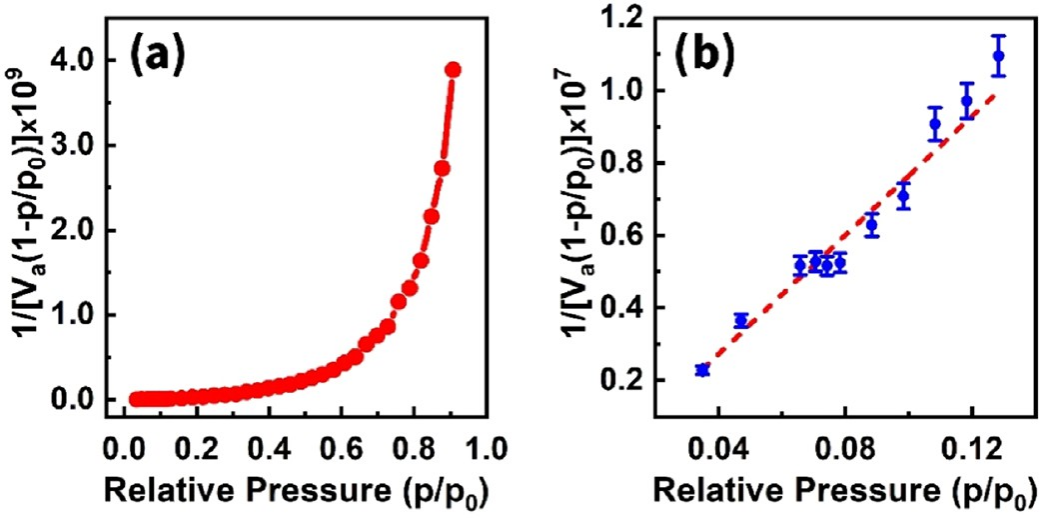
BET plot isotherm (a) and BET linear plot (b).

Once the monolayer volume, *v*_m_, was known, the total surface area can be calculated using eq 10.

10 $$s_{\mathrm{BET}}=\frac{\left(v_{m}\times N\times s\right)}{V\times a}$$

Here, *N* represents Avogadro’s number, *s* is the cross section of the gas molecules, *V* is the molar volume of the adsorbate gas, and *a* refers to the mass of the adsorbent. Thus, BET analysis of the sensor material produced a BET specific surface area (SSA) of 58.06 m^2^/g. Comparing this result with the results of the BET theory available in the literature for pure Fe_2_O_3_, it was found that introducing nanotubes to the surface of the Fe_2_O_3_ led to an incomparably increase in the SSA (Table 3). This confirmed our assumptions that the introduction of nanotubes definitely led to an increase in the effective surface area of the gas-sensitive film and, consequently, an increase in the gas absorption capacity. Besides, based on our experimental results, assuming an average pore volume of 0.175 cm^3^/g, the average pore diameter was estimated to be ∼12 nm. This is a fairly favorable size for the diffusion of ammonia molecules into the gas-sensitive matrix.^87^

**Table 3:** Comparison of the BET Theory Results Available in the Literature between Fe_2_O_3_ and Fe_2_O_3_/MWCNT Materials

material	nanoparticle diameter [nm]	SSA [m^2^/g]	average pore diameter [nm]	ref
α-Fe_2_O_3_	5	24.32	30	^88^
α-Fe_2_O_3_	20	18.31		^89^
Fe_2_O_3_/MWCNT	38.7	53.666	11.07	^90^
Fe_2_O_3_/MWCNT	20–25	58.06	12	this work

## Conclusions

In summary, we presented a highly sensitive NH_3_ gas sensor based on a Fe_2_O_3_/MWCNTs thin film obtained by the e-beam deposition method. The SEM and TEM images of the Fe_2_O_3_ film revealed a granular morphology (with a grain size of 20–25 nm) and 0.5 nm indexed interplanar spacing with the hexagonal α-Fe_2_O_3_ structure, respectively. The tubular appearance of the MWCNTs embedded in each other, with diameters ranging from 80 to 200 nm, and the characteristic interplanar spacing of 0.34 nm as a hexagonal structure were confirmed. The EDX and XPS/FTIR studies of the Fe_2_O_3_/MWCNTs material demonstrated the actual concentration of elements (Fe - 74.62 atom %, O - 20.34 atom %, C - 5.04 atom %), and the types and nature of chemical bonds between the elements, respectively. This sensor exhibited the capability to detect ammonia with a concentration range of 3–650 ppm at 200 °C, corresponding to the response values of 2.7–13.5, respectively. It was confirmed that the introduction of MWCNTs led to an increase in the effective surface area of the Fe_2_O_3_ gas-sensitive film, contributing to the higher gas absorption capacity. Considering its high repeatability, selectivity, long-term stability, and fast response/recovery times (40 and 25 s), we expect that the Fe_2_O_3_/MWCNTs sensor will be considered a prospective gas detector for effective monitoring of environmental ammonia traces.
